# Menorrhagia, Etc., and Hygienics

**Published:** 1888-03

**Authors:** John Meredith

**Affiliations:** Medical Officer of Health, Wellington, Somerset


### MENORRHAGIA, ETC., AND HYGIENICS.

BY

JOHN MEREDITH, M.D. Edin.,

*Medical Officer of Health, Wellington, Somerset.*

---

FOR several years past I have been in the habit of making notes of cases of disordered menstruation that came to me. I carried my inquiries for the purpose into the domestic habits of the patients, the sanitary state of their dwellings—whether exposed to sewer emanations or other noxious influences—the quality of the water drank, and all matters likely to affect health, whether as food, work, rest, or recreation.

I have no reason to suppose that I have had more experience in this form of illness than falls to the lot of any other medical practitioner similarly situated; yet at one time it seemed to me that cases of disordered catamenia were very frequently met with, and by degrees the conviction grew, whilst comparing the history of one illness with that of another, that defective sanitation in some form or other was the main cause, if not the sole cause, in the generality of cases. I exclude cases where uterine tumours or polypi were present.

I found many women peculiarly susceptible to sewer air, and similar effluvia. These persons, when exposed to them, suffered from much loss at the monthly periods; the periods were protracted over many days beyond the usual time, and, soon returning, left but short intervals of freedom. Such patients soon appear anæmic and sallow.

As yet, I have not come across any accounts indicating that polluted water and foul air are causes of menor-

VOL. VI. No. 19.

rhagia; although their influences are now and again in a dim sort of way admitted in cases of amenorrhœa.

The excessive discharge, with its attendant lassitude, seems to me to be only an instance of a blood-poisoning storm running its course—a *zymosis*, parallel to nerve storm—a condition invading nerve-substance, now well recognised among us.

I will submit a few cases by way of illustration. In January, 1883, I attended Mrs. S., age about 40. She complained of severe and prolonged menstrual flow, often passing large clots. She had had a similar attack a couple of months before, which lasted three weeks: the attack in January lasted three weeks also. She was not accustomed to these floodings. Her house at these periods was suffering from a nuisance in the form of sewer gas, which entered the dwelling from the water-closet.

A neighbour of hers, Mrs. C., was affected in a similar way about the same time, and had hæmorrhoids in addition. Her house suffered from an invasion of sewer air, which escaped from a broken pipe in the water-closet. Neither of these women was in the habit of experiencing such excessive catamenial discharge as the ones they then complained of. This only occurred to them, as it afterwards came out, from about the time their closets took to smelling.

After proper representations to the landlord, the closets were put to rights and the nuisance abated, and my two patients again regained their normal habits.

These women were not at all for admitting that the smells had anything to do with their sufferings, as the smells only came at times—not understanding, doubtless, that it is only at times that sewer gas is especially active, depending for this extra activity upon meteorological conditions.

Here is another case, varying a little in character from the foregoing:

Mary C., aged 18, came to see me on the 15th Feb., 1883. She had often been before. She was of a slender frame, and had an anæmic appearance. Her menses recurred every fourteen days—the "red" lasting fully a week, and "whites" for three days more. She was employed at a woollen factory; but her attendance was not regular. She lived at the time with her mother in a house ill-ventilated, devoid of any form of back opening, and it had a close, sour smell. The water which she and her immediate neighbours used was manifestly impure. It was obtained from a well exposed to surface washings and soakage from garden plots in close proximity. M. C. lived with her mother in the same house until December, 1886; and during all the time she suffered as described, no matter what was done. She then removed to another part of the town, to a new house well constructed, and with thorough ventilation. It has also a supply of pure water. Since the change of houses, &c., M. C.'s health has been steadily improving. She does "not see," as she expressed it, "nothing near so much." The courses come on every month, and now only last a few days, and and there are no "whites." She feels herself gaining strength. But she has not yet thrown off the old anæmic, pallid appearance—the result of years' slow poisoning. She is able now to attend to her work at the factory, and does not in any sense consider herself an invalid.

There is another point of interest connected with M. C.'s case, and it is this:

She has a sister, now aged 30, who has been married ten years. This sister, during the time she was at home with her mother, suffered very much in the same way as M. C. did. The catamenia were always profuse. When she married she removed to another part of the town, to a house better ventilated than her mother's, and a fairly good dwelling.

But the water-supply for it was obtained from a well sunk on the lower edge of a cultivated plot of ground in front of the house. The subsoil is sandy, and cultivation extended close to the mouth of the well. Some six years ago I pointed out to the cottagers using this well that the water was not good, as soakage from the garden-plots was entering the well. Nothing, however, was done, as no better water was conveniently available until about a year ago, when a supply of good water, provided by the town, was laid on to the houses, and the old pump has, in consequence, been left severely alone.

During the whole of Mrs. H.'s (M. C.'s sister) married life, up to the beginning of 1887, she suffered from menorrhagia, with the attendant aches and pallor, going from one medical practitioner to another, and deriving at best but temporary relief from any. Once, some seven years ago, as the loss was great, she was told that she must have had a miscarriage. She, however, did not believe this. Anyhow, in the spring of 1887 she felt a change: her health improved, the catamenia ceased, and she became aware that pregnancy had commenced. She, like her neighbours, admitted that her health began to mend when she began using the town water instead of that from the old pump. The supply of good water was not laid on but a few months before the altered condition of things was experienced; and in January of this year Mrs. H. was confined of a son, and all has gone on well.

Many a one, if not most people, I have no doubt, would consider this nothing but a mere coincidence—only a *post hoc*. It conveys to me, at all events, more than that. It has a significance—other things being equal—amounting, practically, to the relationship of cause and effect. Menorrhagia protracted and frequently recurring, as was the case here when the generative organs were in a state of frequent flushing, ought to be considered, if it is not, as a cause of sterility in the female. But, then, what causes the menorrhagia? In this case I contend that it was the impurity circulating in the system; the impure matter being derived from the polluted water which the sufferer freely partook of, and obtained from the well in her garden.

Mrs. A., aged 45, suffered from excessive catamenial discharge during the spring of 1883. The periods lasted fourteen days or more, and the intervals between were brief. She had not, according to her account, suffered from such losses until that year; in fact, not until she removed to her present house, which is a dairy. On visiting the house I perceived a close, heavy odour in it; and this came, as I soon found out, from a manure-heap, placed not far from the back door.

Dairy work was entirely new to Mrs. A., and so was manure smell. The quality of the water obtained from the pump at the back of the house was of a suspicious character, to say the least of it. Added to these, she had the charge for a time of an old pensioner, who was not able to attend to personal cleanliness properly. Observing all these, I ventured to tell my patient and her husband that they were the cause of the illness she was suffering from. Consequently, and as soon as it was practicable, the manure was removed from the back yard, and a fresh and pure supply of water was procured. The result of the removal of the offending influences was a marked improvement in the state of Mrs. A.'s health. The menses resumed their former regular character, and she is now in good health; but the excessive drain of five years ago has left a certain sallowness of countenance. She is, however, free from all organic disorders, and growing stout.

My next illustration is the case of Mrs. F., a lady, age 32. Married several years. No children. Usually healthy and looking well. Not used to have troubles in connection with the catamenia. She came to me once a few years ago, complaining of the menses being excessive; never had such a thing before. Knowing that her house and habits were all that could be desired, I inquired if she had been exposed to some nuisance or other lately; and, after thinking for a while, she admitted that she had been—that her husband was having some drains cleared out near the house, and the smell from them came into the house and made her quite uncomfortable. The nuisance only lasted a day or two.

The treatment resorted to in this and other cases of the kind was, first and foremost, removal of the exciting cause, as soon as this could be ascertained—not an easy matter in many instances—or removal of the patient from exposure to influences which, in my opinion, appeared prejudicial.

In the way of drugs I have used those usually recommended for such cases; namely, cannabis ind., nit. mur. ac. dil., chlorate of potash, ext. ergot, tinct. hamamelis, terebene, &c. Two or more of these, in combination with glycerine, usually give good results for a time; stopping the medicines immediately the flow ceases, and giving iron and quin. sulph. during intervals. But now and then iron could not be tolerated. In such case I began, years ago, to give chlorate of potash, in 12 or 15 grain dose, thrice daily. The result of this was fairly satisfactory. Patients have again and again sent for some of the powders, saying they felt lighter after taking them and could breathe better, as if their blood was better oxydised by the help of the salt; it seemed to supply an element which the stuffy air of their rooms was deficient in, and contribute to the better depuration of their blood, charged by deleterious matter forced into it by unwholesome air and water.

However, this is only by the way. Returning to Mrs. F.'s case. The menorrhagia terminated in about a week's time, and she settled again into her ordinary ways. She has been to me occasionally during late years for similar recurrences, and I always found that the menorrhagia came after exposure to some noxious emanation or other. It might be suggested in regard to this case that the excessive losses were only instances of so many miscarriages. This was the thought that struck me at first; but, while observing their character and the *malaise* accompanying them, I became persuaded that it was not so, and that they were nothing more than so many blood-poison storms expending their force in aggravating the nature of a normal secretion, like a tidal wave.

Visitors at Mrs. F.'s house have had similar experience, but who never suffered in the same way elsewhere.

To go on with the illustrations, here is the case of E. B., age 22, who came to me in August, 1883. She had often been before, complaining of excessive loss during her monthly periods: thought she lost more than double what she ought; and it always lasted over a week: not often free for three weeks at a time; as a rule, her time of freedom was only a little over a fortnight. She "felt out of sorts," had some œdema of her left ankle, and was not fit for her work as a factory hand. Catamenia began with her when she was about fifteen years old, and it has always been excessive. She has always lived in the same house, one of an unhealthy row, without back ventilation in any form, exposed to gutter smells, &c. All the women of the place had a sickly look. At one time pigs were kept in close proximity to the row. The water supplied to the tenants of the row was good, and free from impurity. E. B.'s mother suffered during the "change" period "awfully" from flooding. She is now past it; looking, however, aged and pallid.

Some three years ago the row of houses changed ownership, and the new landlord effected many and much-needed improvements in the sanitary state of the premises; but he was not able to make a through ventilation in the dwellings. Since then E. B.'s health has been much better. The flow is not excessive now. She attends her work regularly, and is seldom at home through illness. There has been no œdema of the foot since the time mentioned.

The story of the next case is something out of the usual course. Mrs. R. F. had been in the habit of coming to see me now and again for years. On the 25th of July, 1883, she came with her youngest son, then twelve years old. The boy has always been mentally defective; cannot be taught in the ordinary way. He can sing, and is possessed of a good deal of mimicry. Evidently he is not one who will be able to take care of himself.

Mrs. R. F., as I have mentioned, had often been to see me on account of obscure ailments—forms of blood-poisoning, as I ventured to consider them. I inquired of her as to the history of her youngest child. She has others, and they are all fairly healthy, with good mental abilities. I cannot hear of any member of the family ever having suffered from mental disorder. The family is, on the father's side, perhaps a little excitable, but not particularly so.

Inquiring of Mrs. R. F. as to her habits, &c., during the time she was in the way with the youngest, she said she had always lived in the same house, was a dyer in a small way, and carried on her work in a small room at the back of the house. I had occasion to examine into the sanitary condition of this dwelling, and found that it had some serious sanitary defects. The drain from the water-closet, situated at the back, passed through the passage, at from one to two feet below the floor, out under the front door to the main sewer in the street. The drain was made of common field-pipes, without sockets and glazing. There was no syphon or any interception between the main and the drain under the house; consequently air from the sewer passed freely into the house drain, and, as it was not efficient, into the dwelling.

Next, as Mrs. R. F. expressed it, "the dye smell was always about." In addition, there was an artificial manure-manufacturing place at the back, only a little outside the premises, the smell from which pervaded her house at times, "so much so that the doors had to be closed against it;" and I suspect that the water used, judging from the position of the well and pump, was not of sound potable quality. Mrs. R. F. was certain that she experienced no shock or fright during the period of her last pregnancy, nor was there any anxiety on her mind; but during the time she was "going" with child she suffered from metrorrhagic discharge. The sanguineous flow continued all the time. She did not think there was one clear week's interval. At times she would fold her arms in cloths wrung out of cold water, as this checked the discharge occasionally. Sometimes big clots would pass. The discharge began, according to her account, one day when she was lifting a heavy garment from her dye vat. She did not know then that she was *beginning*, and only became aware of it afterwards. In common with many others, she used to say that her great "rushes" used to take place in the early mornings. The child when born was weak, and exceptionally feeble during his infancy.

Mrs. R. F. always considered that her sufferings arose from the strain, just mentioned, which she underwent.

Very likely that was the final factor which determined the form of the subsequent morbific action; but if her system had not been in a preternatural condition, prepared by saturation in noxious air and the blood in an impure state, the accidental hæmorrhage would probably not have occurred; or, if induced, the natural repairing power would soon have stopped the leakage, whereas, in Mrs. R. F.'s case, everything seemed to have combined to prevent return to the healthy state.

To my mind, the most interesting part of the case is the character of the child, whose intra-uterine existence was associated with such unusual and depressing circumstances.

It is, as a rule, considered enough, in cases like this, to say that such an one was born an idiot, or mentally defective, and deem it needless to push inquiries further. But when the inquiry is so pushed, and it is elicited that the father or mother had syphilis, or that either or both were drunkards, or that the mother had some distressing shock during the period of gestation—then any such cause is accepted as a matter of course, and deemed final; but when, however, a sufficiently explicit answer in any one of the above lines is not immediately forthcoming, it is not thought unfair to assume the existence of some one of them all the same.

While as to this case there is every reason for believing that the parents were free from all the taints mentioned, and, as stated, there was no shock.

I submit the idea that mental deficiency or deformity may be originated by the mother suffering during pregnancy from uterine hæmorrhage, and breathing in a polluted atmosphere; more as a question for further observations, than as a recognised cause supported by sufficient experience.

*(To be continued.)*

